# Impact of completeness of revascularization by coronary intervention on exercise capacity early after acute ST-elevation myocardial infarction

**DOI:** 10.1186/1749-8090-9-50

**Published:** 2014-03-19

**Authors:** Wei Zhao, Jin Bai, Fuchun Zhang, Lijun Guo, Wei Gao

**Affiliations:** 1 Department of Cardiology, Peking University Third Hospital; Key Laboratory of Cardiovascular Molecular Biology and Regulatory peptides, Ministry of Health; Key Laboratory of Molecular Cardiovascular Sciences, Ministry of Education, Beijing 100191, China

**Keywords:** Myocardial infarction, Percutaneous coronary intervention, Angioplasty, Revascularization, Multivessel disease, Exercise capacity

## Abstract

**Background:**

The importance of achieving complete revascularization by percutaneous coronary intervention (PCI) in patients with acute myocardial infarction (MI) on exercise capacity remains unclear.

**Objective:**

To compare exercise capacity early after acute ST-elevation myocardial infarction (STEMI), in patients receiving PCI with stenting, between those completely revascularized (CR) and those incompletely revascularized (IR).

**Methods:**

We retrospectively reviewed 326 patients [single-vessel disease (SVD) group, 118 patients; multivessel disease (MVD) with CR group, 112 patients; MVD with IR group, 96 patients] who underwent cardiopulmonary exercise testing 7–30 days after STEMI to measure peak oxygen uptake (VO_2peak_), oxygen uptake at anaerobic threshold (VO_2AT_), and peak oxygen pulse. Demographic data, presence of concomitant diseases, STEMI characteristics, and echocardiography and angiography findings were evaluated.

**Results:**

Most patients were male (89.0%) and mean age was 55.6 ± 11.2 years. Ischemic ST deviation occurred in 7.1%, with no significant difference between groups. VO_2peak_ and VO_2AT_ did not differ significantly between groups, despite a trend to be lower in the CR and IR groups compared with the SVD group. Peak oxygen pulse was significantly higher in the SVD group than in the IR group (p = 0.005). After adjustment for age, gender, body mass index, cardiovascular risk factors, MI characteristics and echocardiography parameters, CR was not an independent predictor of VO_2peak_ (OR = −0.123, 95% confidence interval [CI] -2.986 to 0.232, p = 0.093), VO_2AT_ (OR = 0.002, 95% CI 1.735 to 1.773, p = 0.983), or peak oxygen pulse (OR = −0.102, 95% CI −1.435 to 0.105, p = 0.090).

**Conclusion:**

CR in patients with STEMI treated with PCI for multivessel disease might show no benefit on short-term exercise tolerance over IR.

## Background

Primary percutaneous coronary intervention (PCI) in the infarct-related artery (IRA) is now considered the gold standard for patients with acute ST-elevation myocardial infarction (STEMI) [1,2]. Multivessel disease (MVD) is relatively common in patients with STEMI, with a reported prevalence ranging from 50% in highly selected subjects enrolled in randomized clinical trials, such as the CADILLAC study [3], to 80% in those included in more comprehensive registries or those with cardiogenic shock, such as in the SHOCK trial [4,5]. This high frequency raises important therapeutic and prognostic issues concerning whether to target revascularization therapy to the IRA only, or whether to include other vessels affected by stenosis. There are evidences that limiting PCI to the IRA has the advantages of a shorter procedure duration, decreased use of dye, and reduced incidence of peri-procedural myocardial infarction (MI), whereas targeting additional diseased arteries shows the advantages of decreased rate of recurrent angina, and a superior left ventricular ejection fraction (LVEF) [6]. Nonetheless, it is still unclear whether complete revascularization (CR) is superior, equivalent, or inferior to incomplete revascularization (IR), in terms of the risk-benefit balance.

Exercise capacity is a powerful predictor of all-cause mortality [7]. This premise appears to hold true in asymptomatic healthy individuals, as well as in populations of patients with a chronic disease [8]. Recently, several studies demonstrated that cardiopulmonary exercise testing (CPET) is a useful technique for the assessment of myocardial ischemia [9-11], and may be used as a method for measuring the effects of therapy [12,13]. Studies also showed that CPET has a prognosis value in MI patients [7-13].

Available data about the relative advantages and disadvantages of CR and IR in patients with acute STEMI undergoing PCI with stenting is limited. The aim of this study was to use CPET within 30 days of STEMI to assess parameters reflecting exercise capacity (including peak oxygen uptake, VO_2peak_), and to use these as outcome measures to compare the benefits of CR and IR in this cohort of patients. It was predicted that the results of our study would provide additional, useful information that would help guide clinicians making management decisions for patients with MVD requiring revascularization after STEMI.

## Methods

### Study population

The study protocol was conform to the ethical guidelines of the 1975 Declaration of Helsinki, and was approved by the Human Research Committee of the Peking University Third Hospital (Beijing, China), which waived the requirement for informed consent.

We selected our study population from a database, which contained 1600 patients admitted to the Peking University Third Hospital, China, between September 2007 and December 2011 for an acute STEMI. Inclusion criteria were: 1) acute STEMI; 2) successful primary PCI using drug-eluting stents; and 3) CPET performed within 7–30 days of STEMI onset. The clinical criteria used for STEMI diagnosis at the time of admission were: 1) chest pain persisting ≥30 minutes; 2) ST-segment elevation of at least 0.1 mV in ≥2 contiguous electrocardiographic leads; and/or 3) increased levels of cardiac biomarkers (creatine kinase-MB, troponin-I, or troponin-T). Exclusion criteria were: 1) cardiogenic shock at presentation (systolic blood pressure ≤ 90 mmHg); 2) pulmonary edema on admission; 3) previous MI; 4) pulmonary disease comorbidity (such as chronic obstructive pulmonary disease); or 5) previous revascularization procedure (PCI or coronary artery bypass grafting, CABG). After application of these criteria, 326 patients were available for analysis. Patients were included if their whole dataset was available.

Included patients were classified into 3 groups: a single-vessel disease (SVD) group (n = 118), a MVD with CR group (n = 112), and a MVD with IR group (n = 96). Demographic data, comorbidities, STEMI characteristics, angiography findings, revascularization procedure, echocardiography data, and CPET results were evaluated.

### Catheterization protocol

In all patients, diagnostic coronary angiography followed by PCI of the IRA was performed immediately after admission, using standard techniques. PCI of the IRA was based on angiography-guided decisions. The goal of PCI was to restore thrombolysis in myocardial infarction (TIMI) grade 3 flow with residual stenosis <30%. Achievement of this goal was defined as a successful procedure. Delayed PCI of the non-IRA was performed during the subsequent days of the index hospitalization. After primary PCI, all patients received 100 mg of aspirin daily (indefinitely) and 75 mg of clopidogrel daily (for one year), as well as β-blockers (BB), angiotensin-converting enzyme inhibitors (ACEI) or angiotensin receptor blockers (ARB), and statins, if these agents were not contraindicated.

The coronary arteries were classified based on anatomical criteria. The following vessels were considered as major coronary arteries: left main, left anterior descending, circumflex, and right coronary arteries. Diagonal, obtuse marginal, and intermediate branches were considered to be major branches. Lesions at a bifurcation were analyzed as if they were two diseased arteries. MVD was defined as the presence of ≥50% stenosis of ≥2 major epicardial coronary arteries or their major branches, as assessed during initial coronary angiography. The coronary artery disease (CAD) severity in MVD patients was assessed according to a modified Gensini’s stenosis scoring system [14]. This score is computed by assigning a severity score to each coronary stenosis based on the degree of luminal narrowing and its topographic importance. Reduction in lumen diameter was evaluated and assigned a score of 1, 2, 4, 8, 16 and 32 for 25%, 50%, 75%, 90%, 99% and complete occlusion, respectively. A multiplier was also assigned to the value of each affected segment based on the functional significance of the myocardial area supplied by that segment: 5 for the left main coronary artery; 2.5 for the proximal segment of the left anterior descending artery; 2.5 for the proximal segment of the circumflex artery; 1.0 for the right coronary artery, the distal segment of the left anterior descending artery, the posterolateral artery and the obtuse marginal artery; and 0.5 for all other segments.

CR was defined as the absence of total occlusion, with no residual stenosis of >70% found in any major coronary artery or major branch, at discharge, based on visual assessment [15,16]. To validate the visual assessment, quantitative coronary angiography (QCA) was performed in 254 lesions and compared with visual assessment; there was no significant difference between the two methods (P = 0.41, data not shown).

### Cardiopulmonary exercise testing

Current guidelines recommend CPET during hospitalization or early after discharge [17-19]. CPET was performed in our patients with the primary aim of prognosis assessment, and to determine exercise intensity based on VO_2max_ and oxygen uptake at anaerobic threshold (VO_2AT_). After a familiarization test, a symptom-limited CPET was performed within 7–30 days of STEMI, in a single-exercise laboratory. We used a modified Bruce treadmill protocol with continuous monitoring of 12 standard echocardiographic leads. Analysis of ventilatory expired gas was performed using a metabolic system (MAX-II, Physio-Dyne Instrument Corp., USA). The following endpoints were used for termination of the test: 1) achieving 85% of the predicted maximum heart rate for age; 2) respiratory exchange ratio (RER) ≥1.1; 3) presence of chest pain, dyspnea, dizziness, palpitations, leg pain or fatigue; 4) fall in systolic blood pressure of 10 mmHg accompanied by symptoms of lightheadedness; 5) excessive systemic blood pressure increase to ≥230/130 mmHg; 6) sustained ventricular tachycardia or frequent runs of non-sustained ventricular tachycardia; or 7) horizontal or downsloping ST depression ≥0.2 mV. Test results were interpreted by two experienced evaluators who were blinded to: 1) patients’ names; 2) other studies results; 3) patients’ clinical history; and 4) physical findings.

An ischemic ST deviation was defined as ST depression, measured 0.08 s from the J point, of 0.1 mV or more, and followed by more significant ischemia until the end of the test. The definition of VO_2peak_ was the highest O_2_ consumption during any stage of maximal exercise that could be sustained for 30 s. The VO_2AT_ was measured by the V-slope method [20]. The oxygen pulse was defined as the oxygen consumption per heart beat, and is related directly to the stroke volume according to the Fick formula: oxygen pulse = stroke volume x (arterial - venous) oxygen.

### Statistical analysis

Statistical analysis was performed using SPSS 20.0 for Mac (SPSS Inc., USA). Continuous parameters are expressed as mean ± standard deviation (SD) unless otherwise specified; categorical variables are presented as numbers and proportions. For continuous variables, comparisons between groups were made using one-way analysis of variance (ANOVA), with a Fisher’s LSD post-hoc test. Categorical variables were compared using the Chi-square test. Relationships between CPET parameters, angiography findings, subject demographic features, STEMI characteristics, and cardiovascular risk factors were assessed using regression analyses. A p-value <0.05 was indicative of statistical significance.

## Results

### Participants’ characteristics

Clinical characteristics of the participants are summarized in Table 1. The majority of patients were male (89.0%), mean age was 55.6 ± 11.2 years, 48.8% of patients had anterior wall MI, and the proportion of Killip Class I on admission was 97.2%. There were no significant differences between the three groups in any characteristic, with the exception of hypertension: hypertension was more common in both the CR and IR groups than in the SVD group (p < 0.05), but did not differ between the CR and IR groups.

**Table 1:** Participants’ characteristics

	**SVD (n = 118)**	**CR (n = 112)**	**IR (n = 96)**	**p-value**
Age, years	52.7 ± 11.1	57.1 ± 10.9	57.3 ± 11.0	0.912
Male, n (%)	106 (89.8)	102 (91.1)	82 (85.4)	0.401
Hypertension, n (%)	42 (35.6)	54 (48.2)*	57 (59.4)*	0.002
Hyperlipidemia, n (%)	54 (45.8)	59 (56.2)	52 (54.2)	0.334
Diabetes mellitus, n (%)	17 (14.4)	18 (16.1)	23 (24.0)	0.175
Smoking, n (%)	85 (72.0)	80 (71.4)	62 (64.6)	0.052
BMI, kg/m^2^	25.9 ± 3.2	25.4 ± 2.9	26.3 ± 3.0	0.106
Anterior infarction, n (%)	67 (56.8)	53 (47.3)	39 (40.6)	0.059
Killip Class I on admission, n (%)	116 (98.3)	110 (98.2)	91 (94.8)	0.219
Maximum value of CK, IU/L	1421 ± 801	1370 ± 770	1460 ± 879	0.730
LVEDD, mm	49.6 ± 4.5	49.3 ± 6.8	50.3 ± 5.7	0.401
LVEF,%	57.1 ± 8.9	57.3 ± 10.1	55.9 ± 9.0	0.592
Gensini’s score	---	63.2 ± 37.0	65.2 ± 36.5	0.814
IRA				0.080
LM, n (%)	0 (0)	0 (0)	0 (0)	
LAD, n (%)	70 (59.3)	57 (50.9)	38 (39.6)	
LCX, n (%)	16 (13.6)	18 (16.1)	18 (18.6)	
RCA, n (%)	32 (27.1)	37 (33.0)	40 (41.7)	
Medication usage at the time of CPET, n (%)				
RAAS blockers	102 (86.4)	98 (87.5)	88 (91.7)	0.467
ß-blockers	95 (80.5)	80 (71.4)	69 (71.9)	0.207
Statins	101 (85.6)	95 (84.8)	80 (83.3)	0.900

Values are presented as mean ± SD. SVD, single-vessel disease; CR, complete revascularization; IR, incomplete revascularization; BMI, body mass index; CK, creatinine kinase; LVEDD, left ventricular end-diastolic diameter; LVEF, left ventricular ejection fraction; IRA, infarct-related artery; LM, left main coronary artery; LAD, left anterior descending coronary artery;LCX, left circumflex artery; RCA, right coronary artery; CPET, cardiopulmonary exercise testing; RAAS, renin-angiotensin-aldosterone system. *p < 0.05 compared with SVD group.

### Angiography and intervention

Comparison of these data between the IR and CR groups revealed that patients with IR had a higher prevalence of three-vessel disease (57.3 vs. 31.3%, p < 0.001), and were managed using fewer stents (1.6 ± 0.7 vs. 2.1 ± 0.8, p < 0.001) than patients with CR, including the stents from an eventual second PCI in the CR group (Table 1). Extent of baseline stenosis of the non-infarct-related lesions was similar between the CR and IR groups (CR: 83.3% ± 11.7% vs. IR: 81.0% ± 10.8%, p = 0.12). There was no difference in Gensini’s score between the two groups (CR = 63.2 ± 37.0 vs. IR = 65.2 ± 36.5, P = 0.814).

### Stress electrocardiography and cardiopulmonary exercise testing

Results of stress electrocardiography and CPET are shown in Table 2. Ischemic ST deviation occurred in 7.1% of patients, with a peak heart rate of 129.6 ± 18.0 bpm. No significant differences were observed between the CR and IR groups with respect to any of the variables assessed (proportion of patients with ischemic ST deviation, peak heart rate, VO_2peak_, VO_2AT_ and peak oxygen pulse). There were also no significant differences between the MVD groups (IR or CR) and the SVD group with regard to the proportion of patients with ischemic ST deviation, peak heart rate, VO_2peak_ and VO_2AT_. However, there was a trend for VO_2peak_ and VO_2AT_ to be lower in patients with MVD than in those with SVD (Table 2). The peak oxygen pulse was significantly different between the SVD and IR groups (p = 0.005), although no significant differences were observed between the CR group and either the SVD or IR groups.

**Table 2:** Results of the ECG stress test and CPET

	**SVD (n = 118)**	**CR (n = 112)**	**IR (n = 96)**	**p-value**
Ischemic ST deviation, n (%)	6 (5.1)	9 (8.0)	8 (8.3)	0.576
Peak heart rate, bpm	133.6 ± 17.0	128.6 ± 18.2	126.0 ± 18.2	0.283
Vo_2peak_, ml·kg^−1^·min^−1^	23.4 ± 5.5	21.5 ± 5.4	20.1 ± 5.4	0.059
Vo_2AT_, ml·kg^−1^·min^−1^	20.4 ± 4.7	19.0 ± 4.5	19.0 ± 4.8	0.089
Peak oxygen pulse, ml·beat^−1^	13.2 ± 3.3	12.4 ± 3.4	11.9 ± 3.1^*^	0.016

Values are presented as the mean ± SD. SVD, single-vessel disease; CR, complete revascularization; IR, incomplete revascularization; Vo_2peak_, peak oxygen uptake; Vo_2AT_, oxygen uptake at anaerobic threshold. *p < 0.05 compared with SVD group.

### Factors involved in exercise capacity

Stepwise multiple regression analysis was performed to further evaluate the relationship between exercise capacity and the revascularization status of patients with MVD (i.e., those with either CR or IR). In particular, we were interested in determining whether CR, as compared with IR, was a predictor of exercise capacity. The variables selected for the multivariate analysis included age, gender, BMI, hypertension, hyperlipidemia, diabetes mellitus, smoking, anterior wall MI, Killip class I, peak CK value, LVEDD, LVEF, and SVD, CR and IR. After adjustment for age, gender, body mass index (BMI), cardiovascular risk factors, MI characteristics and echocardiography parameters, CR was not found to be an independent predictor of VO_2peak_ (OR = −0.123, 95% confidence interval [CI] -2.986 to 0.232, p = 0.093), VO_2AT_ (OR = 0.002, 95% CI −1.735 to 1.773, p = 0.983), or peak oxygen pulse (OR = −0.102, 95% CI −1.435 to 0.105, p = 0.090). However, as detailed in Table 3, gender, age and LVEF were predictors of VO_2peak_, gender and anterior infarction were negatively correlated with VO_2AT_, and gender, age, BMI and LVEF were associated with peak oxygen pulse.

**Table 3:** Independent predictors of short-term exercise capacity after STEMI

	**OR (95% CI)**	**p-value**
VO_2peak_		
Gender	−3.932 (−6.511 to −1.353)	0.003
Age	−0.092 (−0.167 to −0.017)	0.016
LVEF	0.114 (0.030 to 0.199)	0.008
VO_2AT_		
Gender	−3.428 (−6.755 to −0.102)	0.044
Anterior infarction	−2.155 (−3.874 to −0.436)	0.014
Peak oxygen pulse		
Gender	−3.386 (−4.690 to −2.107)	< 0.001
Age	−0.043 (−0.079 to −0.006)	0.022
BMI	0.405 (0.274 to 0.537)	< 0.001
LVEF	0.086 (0.043 to 0.129)	< 0.001

OR, odds ratio; CI, confidence interval; Vo_2peak_, peak oxygen uptake; LVEF, left ventricular ejection fraction; Vo_2AT_, oxygen uptake at anaerobic threshold; BMI, body mass index.

## Discussion

To the best of our knowledge, the present study is the first to directly compare the effects of CR and IR on CPET carried out within one month in patients with MVD, as a measure of myocardial ischemia. The main findings of our study are that there were no obvious differences in CPET parameters between patients with CR and IR, and that CR was not found to be an independent predictor of these parameters. In addition, the peak oxygen pulse was reduced in patients with IR compared with those with SVD, and there was a tendency for the other CPET parameters to be lower in patients with MVD than in those with SVD. Therefore, we provide novel data showing that CR is not superior to IR in terms of CPET, and conclude that CR may not always offer benefit over IR with regard to the improvement of post-STEMI ischemia. This is of relevance to clinicians making management decisions, and implies that CR should not be an automatic choice for all patients with STEMI and MVD.

The current guidelines from the American College of Cardiology/American Heart Association [18,21] and the European Society of Cardiology [19] are of limited help when it comes to synthesizing the available evidence and informing clinical practice on the issues of MVD in patients undergoing primary PCI. These guidelines state that “although percutaneous coronary intervention in a non-infarct artery is not recommended in stable patients, it can be beneficial in hemodynamically compromised patients if the stenotic artery perfuses a large area of myocardium and the procedure can be performed efficiently.” However, the low level of evidence (level C: “consensus opinion of experts, case studies, or standard of care”) forming the basis of this statement limits the strength of this recommendation.

A recent systematic review and meta-analysis suggested that multivessel PCI in subjects with ongoing or recent STEMI and MVD in non-culprit vessels, although not being associated with a significant increase in adverse events, does not confer any meaningful clinical benefit [22]. Since our CEPT results also suggest that CR does not significantly improve CEPT parameters, we believe it is reasonable to recommend that multivessel revascularization should be only used in patients with instability or very high clinical risk, and that intervention should be deferred in most other subjects until the results of ischemia-proving tests are available. However, another study reported that although multivessel angioplasty during primary PCI for STEMI did not reduce the rate of major adverse cardiac events compared with culprit-vessel-only PCI, CR was associated with a lower rate of repeat revascularization after multivessel PCI [23].

The recent PRAMI trial showed that in patients with STEMI and MVD undergoing culprit artery PCI, preventive PCI in non-infarct coronary arteries with major stenosis significantly reduced the risk of further adverse cardiovascular events, compared with PCI limited to the culprit artery [24]. In contrast, in our study, we conclude that CR may not always offer benefit over IR with regard to the improvement of post-STEMI ischemia. This is of relevance to clinicians making management decisions, and implies that CR should not be an automatic choice for all patients with STEMI and MVD. In the PRAMI study, patients were randomized into two groups, and those with preventive PCI might not undergo CR (like in our study), and those without preventive PCI might otherwise undergo CR [24]. Therefore, the results from the PRAMI trial could not be compared with ours.

It has been suggested that all patients with MVD undergo stress testing shortly after full recovery (or even before discharge) in order to thoroughly appraise the ischemic burden of residual MVD, since it is not uncommon to overestimate the severity of stenosis in non-culprit vessels of patients with STEMI [25]. Electrocardiographic exercise stress testing is an approach that has been proven to be safe and useful after an otherwise uncomplicated MI, and can be performed either before discharge or later [17-19,21].

Still, there is no information concerning the impact of CR after PCI on exercise capacity measured in the early stages (<1 month) after acute STEMI. Although CPET is established as an important diagnostic modality for the clinical assessment of patients with heart failure, its potential utility for defining physiologic abnormalities in other patient populations is being increasingly recognized. Therefore, developing techniques that allow detection of ischemia or disturbances in O_2_ kinetics at an early stage, before the onset of ECG changes and symptoms, will improve diagnostic accuracy to allow suitable intervention before these later effects manifest. Belardinelli *et al.*[10] demonstrated that CPET was able to increase the sensitivity of the standard stress ECG from 46% to 87%, and the specificity from 66% to 74%. The physiologic basis for using CPET gas exchange measurements in patients with coronary artery stenosis was based on their ability to accurately identify the onset of ischemia-induced left ventricular (LV) dysfunction during physical exertion, and to precisely quantify the magnitude of the physiologic impairment [11]. The abnormal physiologic response to CPET may therefore be similar in patients with macro- and micro-vascular ischemia. Recently, a case report described the CPET abnormalities in a patient with suspected micro-vascular coronary artery disease (CAD), and the subsequent improvement in LV function following three weeks of medical therapy with the anti-ischemic drug ranolazine [26]. This would support the use of CPET for the assessment of ischemia severity in patients with CAD.

Our study found that two of the parameters of exercise capacity, namely VO_2peak_ and VO_2AT_, were not significantly different in patients with SVD than in those with MVD, irrespective of whether or not they were completely revascularized. However, there did appear to be a trend for VO_2peak_ and VO_2AT_ to be lower in MVD patients compared with SVD, which may be due to a sample size not large enough to detect this difference. The only significant difference observed was for the peak oxygen pulse, which was higher in the SVD group than in the IR group. It thus remains possible that patients with MVD, particularly those with IR, show impaired exercise capacity as measured using CPET, which may be due to a number of factors. Patients with STEMI are in a heightened thrombotic and inflammatory state and may be more prone to the adverse effects of multivessel PCI [27,28]. Furthermore, multivessel stenting could potentially have adverse effects secondary to side branch closure and distal embolization [29,30]. In addition, it is becoming increasingly recognized that micro-vascular ischemia is a significant cause of exertional intolerance and angina, which can be detected earlier by CPET. The similar performance (in terms of CPET) of patients with CR and IR suggests that CR of macro-vascular stenosis may not be sufficient to restore the blood flow through micro-vessels, resulting in a comparable degree of ischemia.

### Limitations

This study is not without limitations. First, this was a retrospective observational study, rather than a prospective study such as a randomized controlled trial. As a result, we cannot exclude the possibility of a selection bias. Indeed, it is possible that CR was attempted and encouraged in patients in whom it seemed more feasible or more clinically relevant. It is also possible that baseline differences in exercise performance may have existed prior to PCI, and that exercise performance before MI may have been an important determinant of exercise performance shortly after PCI, especially in a non-randomized study, with a small numbers of patients medicated with multiple drugs. Since MI is unpredictable, we could not directly measure pre-MI exercise performance, and would have to rely on indirect measures such as questionnaires, which was not possible in the present study due to its retrospective nature. Second, the amount of ischemia damage was not directly assessed (e.g. using magnetic resonance imaging, MRI), but was measured using Troponin I, which is nevertheless considered gold standard. Third, 97% of our sample had a Killip class 1, indicating that these patients were a low risk population. Achieving a complete CR might show more benefits in higher risk patients. Fourth, the treatment strategy was based on angiography evidences, and not on ischemia evidences. Finally, the choice of endpoints might affect the conclusion. In the present study, we used quantitative endpoints. However, studies using clinical and qualitative endpoints might provide inconsistent results [22]. An appropriately prospectively designed randomized controlled trial, with a larger cohort of participants and with clear measurements of ischemia (such as MRI), would allow for a more detailed assessment of the relative merits of CR and IR in patients with MVD.

## Conclusions

In patients with STEMI and MVD, achieving CR with PCI might not improve exercise capacity. Our results suggest that in patients with STEMI and MVD, attempting CR in all patients may not be the best strategy, and that decisions regarding PCI of the non-infarct vessel(s) may need to be guided by objective evidence of significant residual ischemia. A personalized approach should be advocated. However, further large, randomized trials are needed to confirm these findings.

## Abbreviations

ACEI: Angiotensin-converting enzyme inhibitors; ANOVA: Analysis of variance; ARB: Angiotensin receptor blockers; BB: β-blockers; BMI: Body mass index; CABG: Coronary artery bypass grafting; CAD: Coronary artery disease; CI: Confidence interval; CPET: Cardiopulmonary exercise testing; CR: Complete revascularization; IR: Incomplete revascularization; IRA: Infarct-related artery; LV: Left ventricular; LVEF: Left ventricular ejection fraction; MI: Myocardial infarction; MRI: Magnetic resonance imaging; MVD: Multivessel diasease; OR: Odds ratio; PCI: Percutaneous coronary intervention; SD: Standard deviation; STEMI: ST-elevation myocardial infarction; SVD: Single-vessel disease; TIMI: Thrombolysis in myocardial infarction; VO2AT: Oxygen uptake at anaerobic threshold; VO2peak: Peak oxygen uptake.

## Competing interests

The authors declare that they have no competing interests.

## Authors’ contributions

WZ designed the study, performed CEPT, data collection and statistical analysis, and drafted the manuscript. JB performed CPET and data collection. FZ performed PCI and CPET. LG performed PCI. All authors read and approved the final manuscript.

## References

[B1] Kumar A Cannon CP Acute coronary syndromes: diagnosis and management, part II Mayo Clin Proc 2009 9 11 1021 1036 PubMed PMID: 19880693. Pubmed Central PMCID: 2770915. Epub 2009/11/03. eng 19880693 10.1016/S0025-6196(11)60674-5 PMC2770915

[B2] Lamas GA Escolar E Faxon DP Examining treatment of ST-elevation myocardial infarction: the importance of early intervention J Cardiovasc Pharmacol Ther 2010 9 1 6 16 PubMed PMID: 20061507. Epub 2010/01/12. eng 10.1177/1074248409354600 20061507

[B3] Sorajja P Gersh BJ Cox DA McLaughlin MG Zimetbaum P Costantini C Stuckey T Tcheng JE Mehran R Lansky AJ Grines CL Stone GW Impact of multivessel disease on reperfusion success and clinical outcomes in patients undergoing primary percutaneous coronary intervention for acute myocardial infarction Eur Heart J 2007 9 14 1709 1716 PubMed PMID: 17556348. Epub 2007/06/09. eng 10.1093/eurheartj/ehm184 17556348

[B4] Webb JG Lowe AM Sanborn TA White HD Sleeper LA Carere RG Buller CE Wong SC Boland J Dzavik V Porway M Pate G Bergman G Hochman JS SHOCK Investigators Percutaneous coronary intervention for cardiogenic shock in the SHOCK trial J Am Coll Cardiol 2003 9 8 1380 1386 PubMed PMID: 14563578. Epub 2003/10/18. eng 10.1016/S0735-1097(03)01050-7 14563578

[B5] Chechi T Vecchio S Vittori G Giuliani G Lilli A Spaziani G Consoli L Baldereschi G Biondi-Zoccai GG Sheiban I Margheri M ST-segment elevation myocardial infarction due to early and late stent thrombosis a new group of high-risk patients J Am Coll Cardiol 2008 9 25 2396 2402 PubMed PMID: 18565395. Epub 2008/06/21. eng 10.1016/j.jacc.2008.01.070 18565395

[B6] Jeger RV Pfisterer ME Primary PCI in STEMI--dilemmas and controversies: multivessel disease in STEMI patients. Complete versus Culprit Vessel revascularization in acute ST--elevation myocardial infarction Minerva Cardioangiol 2011 9 3 225 233 PubMed PMID: 21516071. Epub 2011/04/26. eng 21516071

[B7] Kodama S Saito K Tanaka S Maki M Yachi Y Asumi M Sugawara A Totsuka K Shimano H Ohashi Y Yamada N Sone H Cardiorespiratory fitness as a quantitative predictor of all-cause mortality and cardiovascular events in healthy men and women: a meta-analysis JAMA 2009 9 19 2024 2035 PubMed PMID: 19454641. Epub 2009/05/21. eng 10.1001/jama.2009.681 19454641

[B8] Myers J Prakash M Froelicher V Do D Partington S Atwood JE Exercise capacity and mortality among men referred for exercise testing N Engl J Med 2002 9 11 793 801 PubMed PMID: 11893790. Epub 2002/03/15. eng 10.1056/NEJMoa011858 11893790

[B9] Zafrir N Fink G Klainman E Sulkes J Spitzer S Relation between aerobic capacity and extent of myocardial ischemia in patients with normal cardiac function Am Heart J 1999 9 6 Pt 1 1088 1092 PubMed PMID: 10577439. Epub 1999/11/30. eng 10577439 10.1016/s0002-8703(99)70074-8

[B10] Belardinelli R Lacalaprice F Carle F Minnucci A Cianci G Perna G D'Eusanio G Exercise-induced myocardial ischaemia detected by cardiopulmonary exercise testing Eur Heart J 2003 9 14 1304 1313 PubMed PMID: 12871687. Epub 2003/07/23. eng 10.1016/S0195-668X(03)00210-0 12871687

[B11] Chaudhry S Arena R Wasserman K Hansen JE Lewis GD Myers J Chronos N Boden WE Exercise-induced myocardial ischemia detected by cardiopulmonary exercise testing Am J Cardiol 2009 9 5 615 619 PubMed PMID: 19231322. Pubmed Central PMCID: 3035935. Epub 2009/02/24. eng 10.1016/j.amjcard.2008.10.034 19231322 PMC3035935

[B12] Barmeyer A Meinertz T Anaerobic threshold and maximal oxygen uptake in patients with coronary artery disease and stable angina before and after percutaneous transluminal coronary angioplasty Cardiology 2002 9 3 127 131 PubMed PMID: 12417811. Epub 2002/11/06. eng 10.1159/000066320 12417811

[B13] Adachi H Koike A Niwa A Sato A Takamoto T Marumo F Hiroe M Percutaneous transluminal coronary angioplasty improves oxygen uptake kinetics during the onset of exercise in patients with coronary artery disease Chest 2000 9 2 329 335 PubMed PMID: 10936120. Epub 2000/08/11. eng 10.1378/chest.118.2.329 10936120

[B14] Gensini GG A more meaningful scoring system for determining the severity of coronary heart disease Am J Cardiol 1983 9 3 606 PubMed PMID: 6823874 10.1016/S0002-9149(83)80105-2 6823874

[B15] Kalarus Z Lenarczyk R Kowalczyk J Kowalski O Gasior M Was T Zebik T Krupa H Chodór P Poloński L Zembala M Importance of complete revascularization in patients with acute myocardial infarction treated with percutaneous coronary intervention Am Heart J 2007 9 2 304 312 PubMed PMID: 17239694 10.1016/j.ahj.2006.10.033 17239694

[B16] Meliga E Fiorina C Valgimigli M Belli R Gagnor A Sheiban I Resmini C Tizzani E Aranzulla T Scrocca I DE Benedictis M Conte MR Early angio-guided complete revascularization versus culprit vessel PCI followed by ischemia-guided staged PCI in STEMI patients with multivessel disease J Interv Cardiol 2011 9 6 535 541 PubMed PMID: 22010970 10.1111/j.1540-8183.2011.00666.x 22010970

[B17] ATS/ACCP Statement on cardiopulmonary exercise testing Am J Respir Crit Care Med 2003 9 2 211 277 PubMed PMID: 12524257. Epub 2003/01/14. eng 12524257 10.1164/rccm.167.2.211

[B18] Antman EM Anbe DT Armstrong PW Bates ER Green LA Hand M Hochman JS Krumholz HM Kushner FG Lamas GA Mullany CJ Ornato JP Pearle DL Sloan MA Smith SC Jr Alpert JS Anderson JL Faxon DP Fuster V Gibbons RJ Gregoratos G Halperin JL Hiratzka LF Hunt SA Jacobs AK Ornato JP ACC/AHA guidelines for the management of patients with ST-elevation myocardial infarction; A report of the American College of Cardiology/American Heart Association Task Force on Practice Guidelines (Committee to Revise the 1999 Guidelines for the Management of patients with acute myocardial infarction) J Am Coll Cardiol 2004 9 3 E1 E211 PubMed PMID: 15358047. Epub 2004/09/11. Eng 10.1016/j.jacc.2004.07.014 15358047

[B19] Steg PG James SK Atar D Badano LP Blömstrom-Lundqvist C Borger MA Di Mario C Dickstein K Ducrocq G Fernandez-Aviles F Gershlick AH Giannuzzi P Halvorsen S Huber K Juni P Kastrati A Knuuti J Lenzen MJ Mahaffey KW Valgimigli M Van’t Hof A Widimsky P Zahger D Task Force on the management of ST-segment elevation acute myocardial infarction of the European Society of Cardiology (ESC) ESC Guidelines for the management of acute myocardial infarction in patients presenting with ST-segment elevation Eur Heart J 2012 9 20 2569 2619 PubMed PMID: 22922416. Epub 2012/08/28. eng 22922416 10.1093/eurheartj/ehs215

[B20] Beaver WL Wasserman K Whipp BJ A new method for detecting anaerobic threshold by gas exchange J Appl Physiol 1986 9 6 2020 2027 PubMed PMID: 3087938. Epub 1986/06/01. eng 3087938 10.1152/jappl.1986.60.6.2020

[B21] Smith SC Jr Feldman TE Hirshfeld JW Jr Jacobs AK Kern MJ King SB 3rd Morrison DA O'Neil WW Schaff HV Whitlow PL Williams DO Antman EM Adams CD Anderson JL Faxon DP Fuster V Halperin JL Hiratzka LF Hunt SA Nishimura R Ornato JP Page RL Riegel B American College of Cardiology/American Heart Association Task Force on Practice Guidelines; ACC/AHA/SCAI Writing Committee to Update 2001 Guidelines for Percutaneous Coronary Intervention ACC/AHA/SCAI 2005 guideline update for percutaneous coronary intervention: a report of the American College of Cardiology/American Heart Association Task Force on Practice Guidelines (ACC/AHA/SCAI Writing Committee to Update 2001 Guidelines for Percutaneous Coronary Intervention) Circulation 2006 9 7 e166 286 PubMed PMID: 16490830. Epub 2006/02/24. eng 16490830 10.1161/CIRCULATIONAHA.106.173220

[B22] Biondi-Zoccai G Lotrionte M Sheiban I Management of multivessel coronary disease after ST-elevation myocardial infarction treated by primary coronary angioplasty Am Heart J 2010 9 6 Suppl S28 35 PubMed PMID: 21147289. Epub 2010/12/22. eng 21147289 10.1016/j.ahj.2010.10.013

[B23] Jo HS Park JS Sohn JW Yoon JC Sohn CW Lee SH Hong GR Shin DG Kim YJ Jeong MH Chae SC Hur SH Hong TJ Seong IW Chae JK Rhew JY Chae IH Cho MC Bae JH Rha SW Kim CJ Choi DH Jang YS Yoon JH Chung WS Seung KB Park SJ Culprit-lesion-only versus multivessel revascularization using drug-eluting stents in patients with ST-segment elevation myocardial infarction: a Korean acute myocardial infarction registry-based analysis Korean Circ J 2011 9 12 718 725 PubMed PMID: 22259602. Pubmed Central PMCID: 22259602. Epub 2012/01/20. eng 10.4070/kcj.2011.41.12.718 22259602 PMC3257455

[B24] Wald DS Morris JK Wald NJ Chase AJ Edwards RJ Hughes LO Berry C Oldroyd KG PRAMI Investigators Randomized trial of preventive angioplasty in myocardial infarction N Engl J Med 2013 9 12 1115 1123 PubMed PMID: 23991625 10.1056/NEJMoa1305520 23991625

[B25] Hanratty CG Koyama Y Rasmussen HH Nelson GI Hansen PS Ward MR Exaggeration of nonculprit stenosis severity during acute myocardial infarction: implications for immediate multivessel revascularization J Am Coll Cardiol 2002 9 5 911 916 PubMed PMID: 12225715. Epub 2002/09/13. eng 10.1016/S0735-1097(02)02049-1 12225715

[B26] Chaudhry S Arena R Wasserman K Hansen JE Lewis GD Myers J Belardinelli R LaBudde B Menasco N Boden WE The utility of cardiopulmonary exercise testing in the assessment of suspected microvascular ischemia Int J Cardiol 2011 9 1 e7 9 PubMed PMID: 19233492. Pubmed Central PMCID: 3062706. Epub 2009/02/24. eng 10.1016/j.ijcard.2009.01.055 19233492 PMC3062706

[B27] Kornowski R Mehran R Dangas G Nikolsky E Assali A Claessen BE Gersh BJ Wong SC Witzenbichler B Guagliumi G Dudek D Fahy M Lansky AJ Stone GW HORIZONS-AMI Trial Investigators Prognostic impact of staged versus “one-time” multivessel percutaneous intervention in acute myocardial infarction: analysis from the HORIZONS-AMI (harmonizing outcomes with revascularization and stents in acute myocardial infarction) trial J Am Coll Cardiol 2011 9 7 704 711 PubMed PMID: 21816305. Epub 2011/08/06. eng 10.1016/j.jacc.2011.02.071 21816305

[B28] Hannan EL Samadashvili Z Walford G Holmes DR Jr Jacobs AK Stamato NJ Venditti FJ Sharma S King SB 3rd Culprit vessel percutaneous coronary intervention versus multivessel and staged percutaneous coronary intervention for ST-segment elevation myocardial infarction patients with multivessel disease JACC Cardiovasc Interv 2010 9 1 22 831 PubMed PMID: 20129564. Epub 2010/02/05. eng 10.1016/j.jcin.2009.10.017 20129564

[B29] Shishehbor MH Lauer MS Singh IM Chew DP Karha J Brener SJ Moliterno DJ Ellis SG Topol EJ Bhatt DL In unstable angina or non-ST-segment acute coronary syndrome, should patients with multivessel coronary artery disease undergo multivessel or culprit-only stenting? J Am Coll Cardiol 2007 9 8 849 854 PubMed PMID: 17320742. Epub 2007/02/27. eng 10.1016/j.jacc.2006.10.054 17320742

[B30] Varani E Balducelli M Aquilina M Vecchi G Hussien MN Frassineti V Maresta A Single or multivessel percutaneous coronary intervention in ST-elevation myocardial infarction patients Catheter Cardiovasc Interv 2008 9 7 927 933 PubMed PMID: 18798239. Epub 2008/09/18. eng 18798239 10.1002/ccd.21722

